# Impact mechanisms of high-speed railway accessibility on urban expansion in China: An analysis based on the mediating effect model

**DOI:** 10.1371/journal.pone.0357896

**Published:** 2026-10-07

**Authors:** Dan He, Zixuan Chen, Jing Zhou, Yiman Hou, Qimeng Li, Zihang Qin

**Affiliations:** 1 Department of Geography and Urban Science, College of Applied Arts and Science, Beijing Union University, Beijing, China; 2 National Tibetan Plateau Data Center (TPDC), Institute of Tibetan Plateau Research, Chinese Academy of Sciences, Beijing, China; 3 State Key Laboratory of Tibetan Plateau Earth System, Environment and Resources (TPESER), instituteofTibetan Plateau Research, Chinese Academy of Sciences, Beijing, China; University of Salento: Universita del Salento, ITALY

## Abstract

High-speed railway (HSR) has emerged as a transformative force in China, markedly reshaping urban development and spatial structures. Here, we examine the indirect mechanisms through which enhanced HSR connectivity influences urban land expansion. We construct national HSR networks for 2011, 2014, and 2017, and assess city-level accessibility based on network structure, direct connectivity, and service frequency. Urban expansion is measured using the average annual intensity of urban land growth during the periods 2011–2014, 2014–2017, and 2017–2020,taking data from 278 cities in China. Using a mediation and interaction effect models, we explore how HSR accessibility has influenced urban expansion through multiple pathways, including real estate investment, employment density, and industrial restructuring. The results reveal a fundamental paradigm shift: rather than simply stimulating sprawl, high HSR network centrality acts as a “structural brake valve” that significantly inhibits extensive and disorderly outward expansion, driving cities toward compact development. Mechanism analyses indicate that high internal employment density effectively reinforces this centripetal inhibitory effect. Conversely, increased HSR service frequency triggers a strong “masking effect” by stimulating real estate investment, which drives short-term spatial sprawl and obscures the network’s intrinsic compacting tendency. However, interaction models confirm that as the HSR network matures, frequent services combined with capital investment reverse this sprawl, guiding development toward high-density Transit-Oriented Development (TOD).Our findings underscore that HSR has transitioned from a catalyst for urban sprawl to a key driver of intensive, stock-based urban transformation, offering critical policy insights for coordinating transportation infrastructure with sustainable land-use planning and curbing local “land finance” impulses.

## Introduction

China has entered a new era of large-scale high-speed railway(HSR) development. No longer merely a symbol of progress, HSR has become an indispensable element of daily inter-city travel for millions of residents. With the rapid expansion of HSR lines, an increasing number of cities are being woven into the network, continuously transforming its structure and redefining urban accessibility [1–3]. Owing to its exceptional speed, HSR generates a space‒time compression effect that markedly reduces the cost of inter-city travel [4]. Consequently, HSR eliminates barriers to inter-city travel, broadens the travel horizons of residents, augments the frequency of inter-city journeys, and ultimately alters mobility patterns [5,6]. Changes in inter-city mobility resulting from HSR development potentially influence urban economic performance, while simultaneously fostering economic integration among cities along HSR corridors [7,8]. Such shifts in urban economies and demographic dynamics typically precipitate alterations in land use [9], and improvements in the accessibility of cities and HSR stations stimulate the economic development of cities. Therefore, HSR exerts a profound impact on land use efficiency and, in particular, on urban expansion [10].

Many countries, including China, are undergoing rapid urbanization. In China alone, the area of urban land expanded fourfold between 1990 and 2020. The continuous expansion of urban land is essential to meet the space demands of both the residential and industrial sectors, as well as to achieve the objective of economic growth. However, this expansion inevitably encroaches upon cultivated land, vegetation and other land types, which may have profound implications for the urban ecological environment. Such land transformations often result in a range of urban challenges, including environmental degradation and land-use conflicts. Consequently, understanding the dynamics of urban expansion has emerged as an important research focus in urban sustainability studies. Population growth, economic development, industrial structure and transportation development are particularly influential drivers of urban expansion [11]. In particular, transportation accessibility is widely recognized as a fundamental driving force of urban expansion, because changes in accessibility affect location choices, redistributing economic activities and production factor inputs [12]. High-speed railway is regarded as a transformative force in redefining urban accessibility. Consequently, growing research attention has been directed toward investigating the relationship between HSR development and urban expansion. Current research in this domain primarily focuses on the impact on urban expansion of opening new HSR lines, the frequency of HSR services, and the location of HSR stations in cities. Such studies indicate that both the opening of a new HSR line and its service frequency exert a substantial influence on urban expansion [13–15]. Moreover, the opening time of HSR significantly affects the speed of urban land-use conversion and accelerates the loss of agricultural land [16].

Although He et al. [17] explored the characteristics and impacts of the HSR network on the urban structures of major urban agglomerations within China, several knowledge gaps within this field remain. First, previous studies have neglected the perspective of the HSR network when examining the impact of HSR accessibility on urban expansion. Cities are places in which various flow factors are exchanged; the urban spatial structure is reshaped by flow factors, and the land-use system undergoes corresponding changes [18]. In this context, the HSR network intensifies the movement of factors between cities, links resources that are otherwise scattered, and facilitates resource sharing and collaborative efforts among urban centers [19]. The accessibility of cities in the HSR network represents the ability of cities to gather dispersed resources and exert spillover effects [19], potentially affecting the intensity of urban expansion. However, the current literature remains limited in its exploration of this particular influence. Second, there is a notable dearth of empirical research on the impact mechanism(s) of HSR accessibility in relation to urban expansion. Existing studies predominantly rely on statistical analyses and theoretical models to explain the effects of HSR introduction and general accessibility, but they frequently overlook empirical validation of the socio-economic pathways mediating this relationship. This oversight is critical because, beyond its direct effects, HSR accessibility may also indirectly drive urban expansion by modulating other pivotal variables.

Here, we aim to bridge the aforementioned knowledge gaps. First, we construct a comprehensive national HSR network and interpret four accessibility indicators(closeness centrality (CC), betweenness centrality (BC), degree (DG), and the frequency of HSR stops in a city (FRE) for each city within the network to explore the impact of HSR accessibility on urban expansion. Second, we build an mediating effect model, and select the mediating variables of employment density (EMP), investment in real estate development (IRE), and proportion of added value of the tertiary industry (TER) to explore the impact mechanism of HSR accessibility on urban expansion. The core problem we aim to solve is to unravel the mechanism by which HSR accessibility drives urban expansion. Our results are expected to provide valuable insights for governmental bodies in forecasting future trends in urban land use and facilitating refined management in the era of the HSR network. Furthermore, this study offers practical guidance for land management and urban planning departments to optimize urban growth boundaries and effectively regulate spatial expansion, facilitating a strategic transition from extensive land consumption toward sustainable, compact urban development.

### Reviewing the impact mechanism of HSR on urban expansion

The proliferation of HSR networks has emerged as a critical driver of urban spatial expansion, operating through interconnected demographic, economic, and related channels. Accessibility improvements induced by HSR development initiate a cascade of spatial‒economic transformations. Reduced inter-city travel costs and time compression effects fundamentally alter the mobility patterns of various factors, enabling accelerated flows of labor, capital, and technology across city networks [20,21].

High-speed railway facilitates urban expansion by attracting people to cities. The introduction of HSR and enhancement of service frequency have markedly reduced travel time and expenses, thereby augmenting urban accessibility and competitiveness [13,14,22]. This improved transportation network facilitates the agglomeration of both population and commercial activities, thus generating additional employment opportunities [23]. In turn, these improved employment prospects draw labor from surrounding regions to cities served by HSR, further driving urban development and expansion [5,11,24]. The resulting concentration of population increases the demand for housing and public services, thereby stimulating real estate development and infrastructure construction, which ultimately stimulates further urban expansion [14,25]. Population agglomeration increases the cost of living in city centers causing the population to further concentrate in the suburbs, where land prices are lower. Ultimately, this increases the demand for housing and drives urban expansion in the suburbs [16].

In addition to fostering population agglomeration, HSR can also promote industrial agglomeration. High-speed railway improves inter-regional accessibility, strengthens urban influence, expands the urban service radius, and integrates internal and external markets, expanding market size and bringing greater market potential. These factors collectively enhance urban competitiveness, attracting companies and fostering the formation of industrial clusters [13,16,26]. The time‒space compression effect of HSR contributes to economies of scale and reduces production costs, thereby reducing transaction and communication expenses. This, in turn, enhances the movement of essential production factors, including labor, capital, and technology [24]. In this way, the reduction in transaction costs further reinforces industrial agglomeration [16,27]. High-speed railway networks connect resources dispersed across different cities; and the accessibility of a city in the HSR network represents its capacity to concentrate resources and generate spillover effects [19]. As the HSR network continues to evolve, increasing urban accessibility strengthens inter-city synergy and integration [28], This process fosters investment agglomeration, enhances borrowed size effects, and ultimately promotes industrial agglomeration [19]. Industrial agglomeration increases the demand for urban construction land [13], promotes real estate investment, and consequently results in urban expansion [5].

Structural economic transformations further compound urban expansion. High-speed railway strengthens inter-city flows of production factors [29,30]; notably, the production factors and products of the secondary and tertiary industries have higher mobility than those associated with primary industry [13]. This mobility differential drives structural transformation towards non-agricultural industries, accelerating the conversion of agricultural land to urban uses and stimulating metropolitan expansion. Service industries particularly benefit from HSR-induced agglomeration economies, resulting in increased service sector shares at the expense of manufacturing [31]. Manufacturing industries exhibit greater cost sensitivity [31]; hence, the industrial agglomeration caused by HSR development increases the pressure of competition, with the market crowding effect forcing companies to choose locations with fewer competitors [16]. This dynamic often directs manufacturing firms towards the urban periphery. Consequently, for HSR stations located in suburban areas, lower factory construction costs in these locations serve as a strong incentive for manufacturers to establish operations nearby, capitalizing on infrastructure advantages while optimizing investment returns [24]. In short, HSR development directly results in the conversion of agricultural land into urban land through the upgrading of industrial structure. Meanwhile, the expansion of the service sector necessitates the development of complementary real estate and infrastructure. The increasing land costs brought about by agglomeration further promote the spatial relocation of manufacturing and drive urban land expansion in suburbs [18].

Underpinning these phenomena lies the capacity of HSR to reconfigure spatial‒economic equilibria. High-speed railway promotes urban expansion through economic growth channels, including improved investment efficiency, enhanced enterprise production efficiency, and increased household consumption. It also reduces travel costs and fosters the agglomeration of production factors, such as industry and labor force. This generates agglomeration benefits and also creates a knowledge spillover effect, facilitating knowledge exchange and mutual learning among companies. Consequently, this enhances the innovation capacity and productivity of enterprises and improves the overall competitiveness of cities [31–33]. The cost-reduction effects induced by HSR enable firms to pursue specialized investments across cities, enhancing regional labor division and industrial productivity through strategic market segmentation. Improved inter-city connectivity facilitates rapid information acquisition and face-to-face negotiations between investors and policymakers, significantly optimizing capital allocation and operational decisions [34]. Hence, HSR enables enterprises to reduce investment costs, improve decision-making efficiency, attract more high-quality investment, and improve urban production efficiency [33]. Time savings induced by HSR expand the leisure mobility of residents, stimulating cross-city consumption patterns [13,35] that drive the development of consumer service industries through scale effects [26,36]. Ultimately, HSR promotes the development of the urban economy by boosting consumption vitality and production efficiency, then accelerates the demand for urban construction land and the intensity of urban expansion [12,13].

Theoretically, HSR fosters urban expansion by enhancing accessibility and the inter-city flow of production factors. However, although the link between HSR and urban growth is well-established [12,13], the internal transmission mechanisms remain underexplored. The existing literature emphasizes pro-growth effects but largely neglects the ways in which HSR-induced sectoral shifts might also inhibit or reshape urban expansion. This oversight obscures the dual-faceted nature of the impact of HSR, creating a critical knowledge gap.

A new perspective on this problem is offered by complex network theory, which uses metrics such as centrality and degree to correlate the network position of a city with its development [17,19,21,37]; however, the underlying logic remains a “black box.” The specific economic channels, their relative intensity, and the conditions under which network connectivity translates into physical land expansion still require rigorous, model-based validation [38].

To address these knowledge gaps, we here develop a mechanism-oriented framework. We employ a mediation effect model that integrates HSR network indicators with key economic and demographic variables to systematically test the direct and indirect pathways from network connectivity to urban expansion, thereby quantifying the strength and direction of these transmission mechanisms. Ultimately, this research provides crucial theoretical insights and empirical evidence to support more nuanced and effective coordinated transportation and land-use policies in the context of rapid urbanization.

## Data and methods

### Measurement of urban expansion

The average annual expansion intensity of urban land (AUEI) represents the percentage of the average annual growth of urban land within the administrative area of each city over a specified period. The AUEI index is utilized to illustrate the differences in the expansion intensity of urban land [39].


$$\begin{array}{c} AUEI=(U_{end}-U_{start})/\left(U_{urban}\right)/N\times 100\%, \end{array}$$ (1)

where *U*_*start*_ and *U*_*end*_ represent the urban land area of each city at the beginning and end of the study respectively. *U*_*urban*_ represents the administrative area of each city. *N* represents the number of years during the study period.

### Interpretation of urban multi-dimensional HSR accessibility

The Chinese HSR network in this study was constructed using the HSR network and city node data from 2011, 2014, and 2017, with the direct HSR frequency between cities serving as the weight of the network, and each HSR city acting as a node, the HSR networks and HSR flows for 2011, 2014, and 2017 are shown in Fig 1 and Fig 2. And four indicators representing multi-dimensional HSR accessibility of each city in the network (closeness centrality (CC), betweenness centrality (BC), degree (DG), and weighted degree (WD)) were calculated. The four centrality indicators all focus on HSR accessibility, but with different emphases. CC reflects “time-space compression accessibility” and indicates the average shortest travel time from the city to others. DG represents “direct connectivity accessibility” and characterizes the number of cities with direct HSR links. WD integrates HSR service frequency to form “high-efficiency service accessibility” and more accurately reflects actual accessibility levels. BC belongs to “transfer hub accessibility” and highlights the city’s key role in allocating interregional resources, complementing the other three indicators.

**Fig 1:**
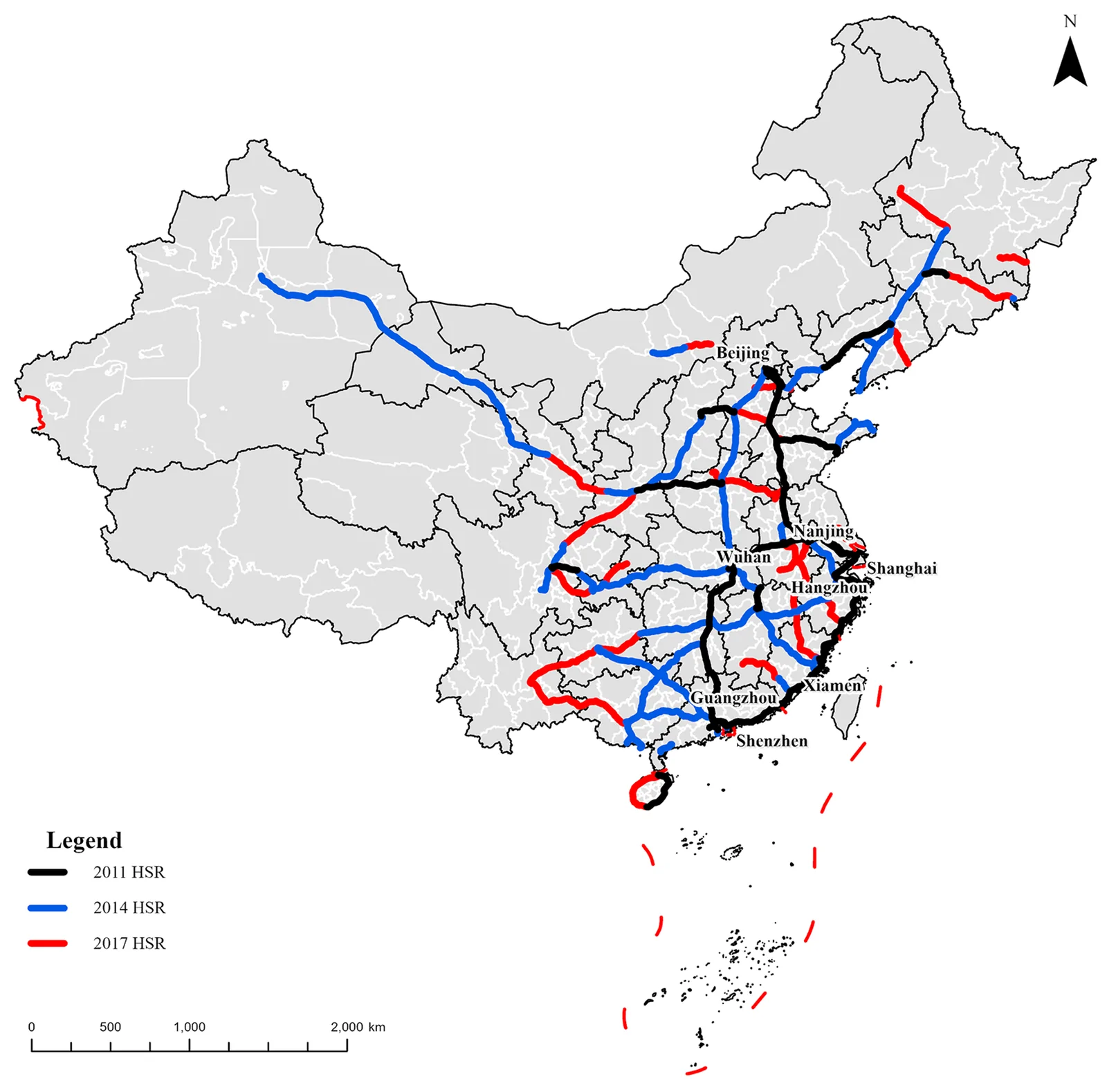
China’s HSR network in 2011, 2014, and 2017.

**Fig 2:**
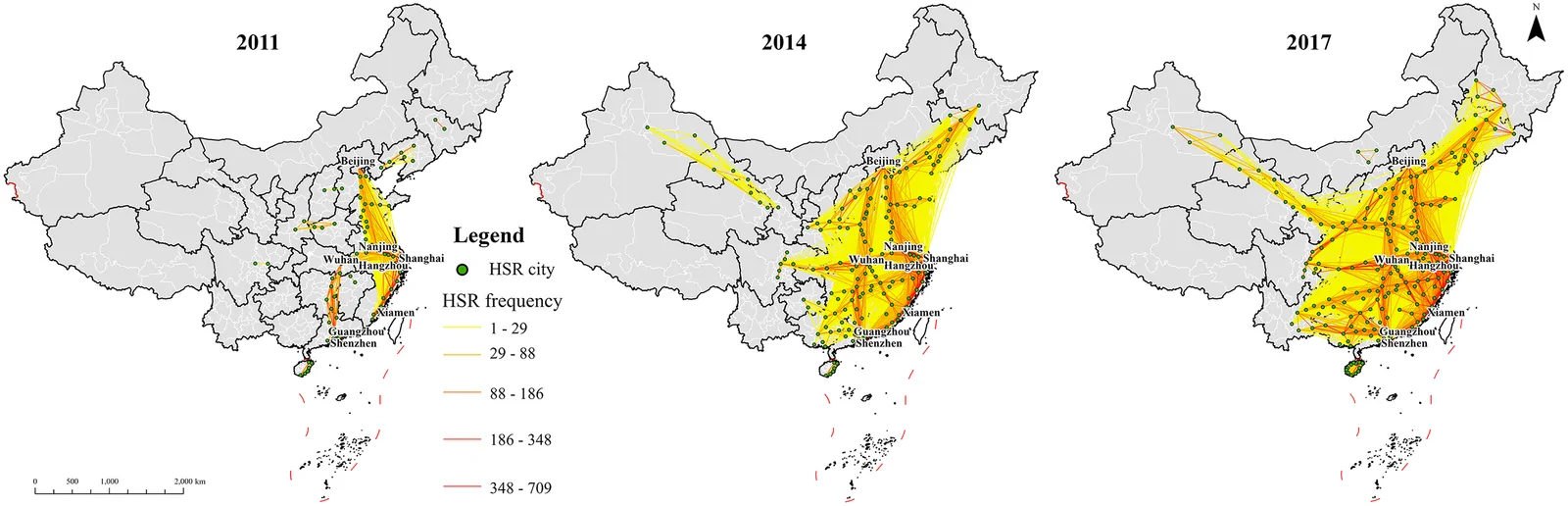
China’s HSR flows in 2011, 2014, and 2017. Base map as in Fig 1.

Administrative boundary data were obtained from the 2024 national provincial-, prefectural-, and county-level administrative division dataset of China provided by the National Geomatics Center of China through Tianditu (Map Approval No. GS(2024)0650).

### Closeness centrality

Closeness centrality measures the extent to which a node is close to all other nodes along the shortest paths, reflecting its accessibility within a given network. The CC of a node is calculated as the inverse of the average shortest distance from that node *i* to all other nodes in a given network. A larger CC value indicates greater convenience in reaching other nodes [40]. In a HSR network, a higher CC signifies that the city of interest is more accessible to other cities.


$$\begin{array}{c} C_{C}\left(i\right)=\frac{\text{n-}1}{\sum_{v_{j}\in V,i\neq j}d_{ij}}, \end{array}$$ (2)

where *d*_*ij*_ represents the shortest path between nodes *i* and *j*; *V* = {v_i_: i = 1, 2,···, n}, *n* = |V|, and *n* is the number of nodes.

### Betweenness centrality

Betweenness centrality is defined as the probability that a node lies on the shortest path between two other nodes. This index reflects the probability of a city serving as a crucial transfer point between two other cities that are not directly connected, highlighting its mediating role and control within the HSR network [40]. A higher city BC value of a city indicates that the city is located on the shortest paths connecting numerous city pairs; this demonstrates a significant transit impact and control capacity, as well as high accessibility.


$$\begin{array}{c} C_{B}\left(i\right)=\sum_{k\neq i\neq j\in N}\sigma_{kj}\left(i\right)/\sigma_{kj}, \end{array}$$ (3)

where *C*_*B*_*(i)* represents the BC of node *i*; $\sigma_{\text{kj}}$represents the sum of shortest paths between nodes *k* and *j*; and $\sigma_{\text{kj}}\left(i\right)$represents the number of shortest paths from node *k* to *j* through node *i*.

### Degree

The DG of a node is defined as the number of edges directly connected to that node within a complex network. In the HSR network, the DG of a city represents the number of cities directly connected to it by HSR. A higher DG indicates greater accessibility, meaning that more cities can be reached directly via HSR from the city of interest.


$$\begin{array}{c} DG_{i}=\sum_{j=1,j\neq i}^{n}L_{ij}, \end{array}$$ (4)

where *DG*_*i*_ represents the DG of city *i*, *L*_*ij*_ represents the number of sides directly connected between city *i* and city *j*, *L*_*ij*_ = 1 indicates that there is a direct HSR link between *i* and *j*, *L*_*ij*_ = 0 indicates that there is no direct HSR link between *i* and *j*; and *n* indicates the total number of cities.

### Weighted degree

The DG examines the accessibility of each city within the HSR network by assessing the presence of direct HSR connections between cities, but neglects the frequency of HSR services. In contrast, the WD metric accounts for the existence of direct HSR links and also incorporates the weight of the HSR network, specifically the frequency of HSR services between cities. A higher WD indicates a greater frequency of direct HSR connections between the city of interest and other cities, thereby providing a more comprehensive reflection of the HSR accessibility of a given city.


$$\begin{array}{c} WD_{i}=\sum_{j=1,j\neq i}^{n}w_{ij}, \end{array}$$ (5)

where *WD*_*i*_ represents the WD of city *i*, *w*_*ij*_ represents the frequency of direct HSR services between city *i* and city *j*; and *n* represents the total number of cities.

### Mediating effect model

The HSR accessibility not only has a direct influence on the intensity of urban expansion, it also has an indirect affect through socioeconomic variables. These socioeconomic variables serve as mediating factors. Hence, the construction of a mediating effect model is essential to understand the impact mechanism(s) of HSR accessibility on urban expansion. Referring to previous literature [19,41–43], equations for the mediating effect model are presented as follows:


$$\begin{array}{c} Y_{it}=\beta_{\text{0}}+cX_{it}+\gamma Controls_{it}+\mu_{i}+\lambda_{t}+\varepsilon_{it} \end{array}$$ (6)

$$\begin{array}{c} M_{it}=\beta_{\text{0}}+aX_{it}+\gamma Controls_{it}+\mu_{i}+\lambda_{t}+\varepsilon_{it} \end{array}$$ (7)

$$\begin{array}{c} Y_{it}=\beta_{\text{0}}+bM_{it}+c^{\prime }X_{it}+\gamma Controls_{it}+\mu_{i}+\lambda_{t}+\varepsilon_{it} \end{array}$$ (8)

Equations (7) and (8) together constitute the test model for the mediating effect. Where: the subscripts i and tdenote the city and year, respectively; $Y_{it}$is the urban land expansion intensity (AUEI);$X_{it}$represents the multidimensional indicators of HSR network accessibility (CC, BC, DG, FRE); $M_{it}$represents the mediating variables (EMP, IRE, TER); and $Controls_{it}$ denotes the set of control variables. $\beta_{\text{0}}$ is the constant intercept term; γis the coefficient vector for the control variables; $\mu_{i}$represents the city fixed effect, capturing time-invariant, unobservable city-specific characteristics; $\lambda_{t}$ represents the time fixed effect, accounting for macroscopic shocks common to all cities in specific years; and $\varepsilon_{it}$ is the random error term. The coefficient *c* represents the total effect of the explanatory variable on the dependent variable (AUEI). The coefficient *a* represents the effect of the explanatory variable on the mediating variable, while the coefficient *b* represents the effect of the mediating variable on the dependent variable after controlling for the explanatory variable. The coefficient *c’* represents the direct effect of the explanatory variable on the dependent variable after controlling for the mediating variable. The relationship can be expressed as *c = c’ + ab*, where *ab* reflects the indirect effects of the explanatory variables on the dependent variables [41].

To eliminate complex bias interferences caused by random noise, intercept terms, and spatiotemporal heterogeneities in the original algebraic expansion, this model introduces city fixed effects and time fixed effects. By setting a zero intercept and following the statistical principle that the mean of residual terms is zero, the analysis focuses on the path coefficients that reflect marginal changes between variables [43], thereby ensuring a strict additive equivalence relationship between the total effect, direct effect, and indirect effect ($c=c^{\prime }+ab$), laying a solid econometric theoretical foundation for subsequent parameter estimation and significance testing of effect values. Chang (2022) used a mediating effects model to analyze the impact of digital finance on agricultural carbon emissions, which supports the feasibility of our model design [42]. Indirect effects encompass both mediating and suppressing effects. The conceptual figure of the mediating-effect model is shown in Fig 3.

**Fig 3:**
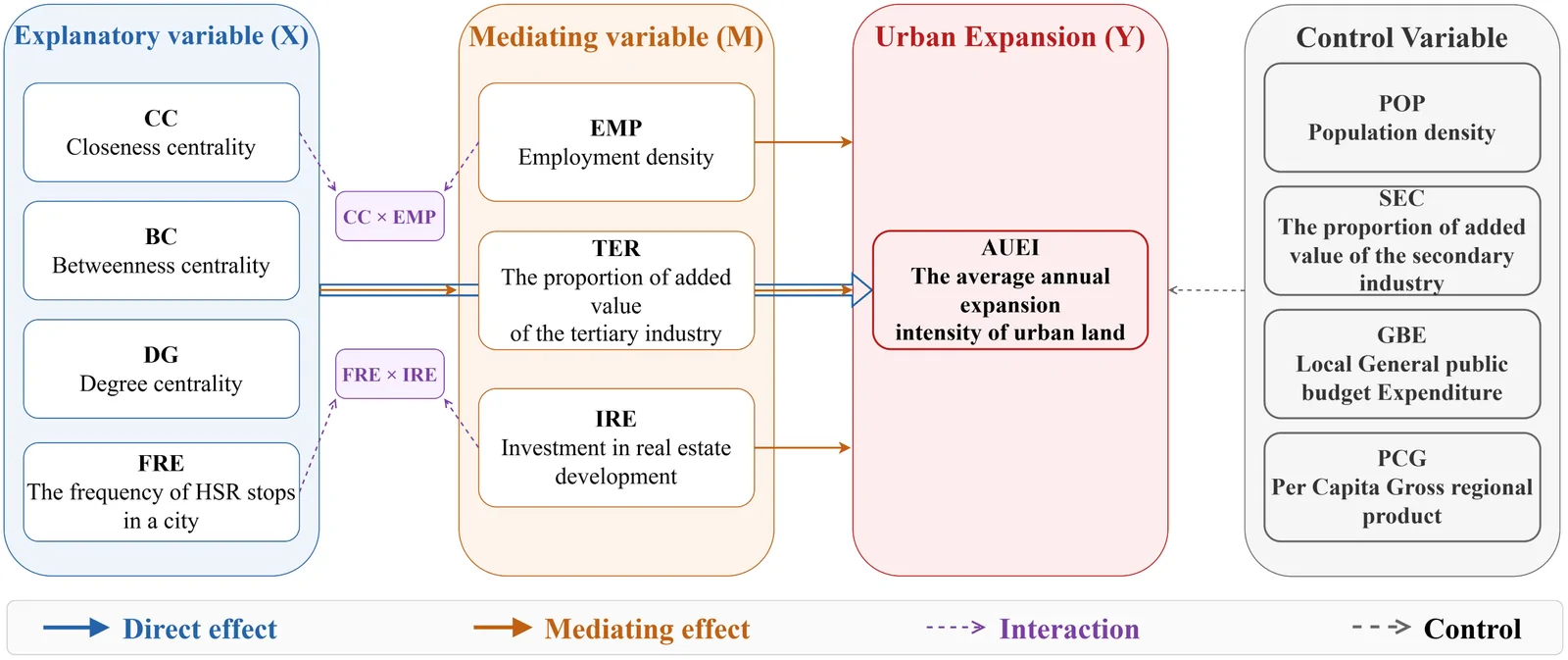
Mediating-effect model conceptual figure.

The analysis flow is as follows [44]:

Step 1: The coefficient *c* of Eq. (6) is checked. If it is significant, the analysis can proceed to the next step; if not, the result is discussed in terms of a suppressing effect.

Step 2: The coefficients *a* of Eq. (7) and b of Eq. (8) are tested successively. If both coefficients are significant, the indirect effect is considered significant, and the analysis proceeds to Step 4. If at least one of the coefficients is not significant, the analysis proceeds to Step 3.

Step 3: The bootstrap method is used to test the significance of *ab*. This method works by repeatedly resampling the dataset (e.g., 5,000 times) to estimate the distribution of ab. On this basis, a confidence interval is calculated. If the confidence interval does not include zero, the indirect effect is significant. Compared with traditional methods such as the Sobel test, the bootstrap method is more accurate and reliable, especially when the sample size is relatively small or the data distribution is non-normal. If the result is significant, the analysis continues to Step 4. If not, it stops because there is no evidence of a meaningful indirect effect. Relevant references are given here to support this approach [41,45].

Step 4: The coefficient *c’* of Eq. (8) is checked. If it is not significant, meaning that the direct effect is not significant, this indicates that there is only a mediating effect. If it is significant, indicating that the direct effect is significant, the analysis proceeds to the Step 5.

Step 5: The signs of *ab* and *c’* are compared. If the two signs are the same, a mediating effect is indicated, and the proportion of the mediating effect in the total effect *ab/c* is reported. If the signs are different, a suppressing effect is indicated, and the absolute value of the proportion of the suppressing effect to the direct effect *|ab/c’|* is reported.

### Variable summary and data sources

In addition to assessing the HSR accessibility of each city within the HSR network, the frequency of HSR stops in a city is also considered as a measure of HSR accessibility. According to existing literature, the accessibility advantages provided by HSR have potential impacts on real estate development, residential consumption, population and labor distribution, and various industries [27‒31]. These factors may also influence urban expansion; hence, it is inferred that HSR will affect the intensity of urban expansion by influencing the aforementioned factors. High-speed railway accessibility is taken as the explanatory variable, while EMP, IRE, and TER are taken as mediating variables. Other socioeconomic factors, including POP, SEC, GBE, and PCG, are incorporated as control variables. This is summarized in Table 1. Table 2 details the key descriptive statistics of the four HSR network centrality metrics and the AUEI variable utilized in this study.

**Table 1:** Summary of variables.

Variable type	Variable name	Variable abbreviation	VIF
Explanatory variable	Closeness centrality	CC	1.58778
	Betweenness centrality	BC	2.05159
	Degree	DG	2.50405
	Weighted degree	WD	
	The frequency of HSR stops in a city	FRE	3.34319
Mediating variable	Investment in real estate development	IRE	5.12999
	Employment density	EMP	1.89441
	The proportion of added value of the tertiary industry	TER	4.61774
Control Variable	Total retail sales of consumer goods	RSG	
	Population density	POP	1.09615
	The proportion of added value of the secondary industry	SEC	3.59116
	Local General public budget Expenditure	GBE	4.22849
	Per Capita Gross regional product	PCG	3.14236

**Table 2:** Descriptive statistical results of HSR network centrality and AUEI.

Variable	Obs.	Mean	S.D.	Min.	Max.
CC	418	5.11633	1.50461	0.476	6.161
BC	418	61.13123	115.5172	0	1015.301
DG	418	46.99761	34.29575	1	147
WD	418	1425.768	1451.314	13	8422
AUEI	418	0.007243	0.006622	6.01E-06	0.036148

The HSR frequency between cities and the frequency of HSR services within city were collected and summarized from the Shengming and Jipin timetables, which are commonly used applications in China for checking train schedules, ticket prices, and service frequencies. Compared with the official China Railway 12306 website, these applications have the advantage of allowing users to access historical train- schedule data. The IRE, RSG, SEC, and TER data were derived from the China City Statistical Yearbook, while POP data was derived from the China Urban Construction Statistical Yearbook. The EMP data was calculated as the number of employed people/area of administrative units, with data derived from the China City Statistical Yearbook. The time points at which the aforementioned data were obtained were 2011, 2014, and 2017, covering 278 cities with HSR stations or direct HSR connections. while AUEI values were calculated for the periods 2011‒2014, 2014‒2017 and 2017‒2020. This approach accounted for the time lag associated with changes in urban expansion intensity resulting from changes in HSR accessibility. Urban land-use data for 2011, 2014, 2017, and 2020 were sourced from a 30-m annual land-cover dataset published in Earth System Science Data [46]. The 278 cities were selected based on two criteria: (1) having at least one HSR station in the administrative area by the end of the study period; (2) having complete urban land-use data and socioeconomic data from official statistical yearbooks. Cities without HSR access or with missing core data were excluded to ensure the reliability of the mediating effect analysis.

First, the Variance Inflation Factor (VIF) of each variable was tested. Among the explanatory variables, WD is defined as in Equation (5). WD (weighted degree centrality) is a composite index derived from HSR service frequency. When analyzed together with FRE (stop frequency), it inherently introduces a high degree of mathematical redundancy. Compared to WD, FRE can more directly reflect the intensity of operational services and helps to more clearly decompose the network breadth (DG) and operational density. Among the control variables, the indicator of RSG highly overlaps with PCG and GBE, and acting as a control variable would affect the subsequent operation of the model. Therefore, when WD is eliminated and the remaining four variables related to HSR accessibility (CC, BC, DG, and FRE) are retained as explanatory variables, and RSG is eliminated and the remaining four variables related to city indicators (POP, SEC, GBE, and PCG) are retained as control variables, all VIF values are less than 10, indicating that there is no multicollinearity (see Table 1).

This study utilizes panel data from multiple prefecture-level cities in China across three time periods. To examine the correlation between explanatory variables and individual effects, thereby making a scientific choice between the fixed effects model and the random effects model, this study conducts a Hausman test on the mediating effect model. The test results in Table 3 indicate that the Chi-square statistics for the four displayed variable combinations exhibit high significance (p = 0.000), rejecting the null hypothesis. This indicates the presence of endogeneity problems, so the subsequent empirical analysis uniformly adopts the two-way fixed effects model.

**Table 3:** Hausman Test.

Variable	Chi-square	p-value	Choice
CC-EMP-AUEI	69.48	0.000	FE
BC-IRE-AUEI	89.22	0.000	FE
DG-TER-AUEI	166.981	0.000	FE
FRE-EMP-AUEI	330.30	0.000	FE

To intuitively character the structural dynamics of the HSR infrastructure and urban development, Figs 4 and 5 map the spatiotemporal evolution of closeness centrality (CC), betweenness centrality (BC), degree centrality (DG), weighted degree (ED) and the average annual expansion intensity of urban land (AUEI) across 278 Chinese cities in 2011, 2014, and 2017.

**Fig 4:**
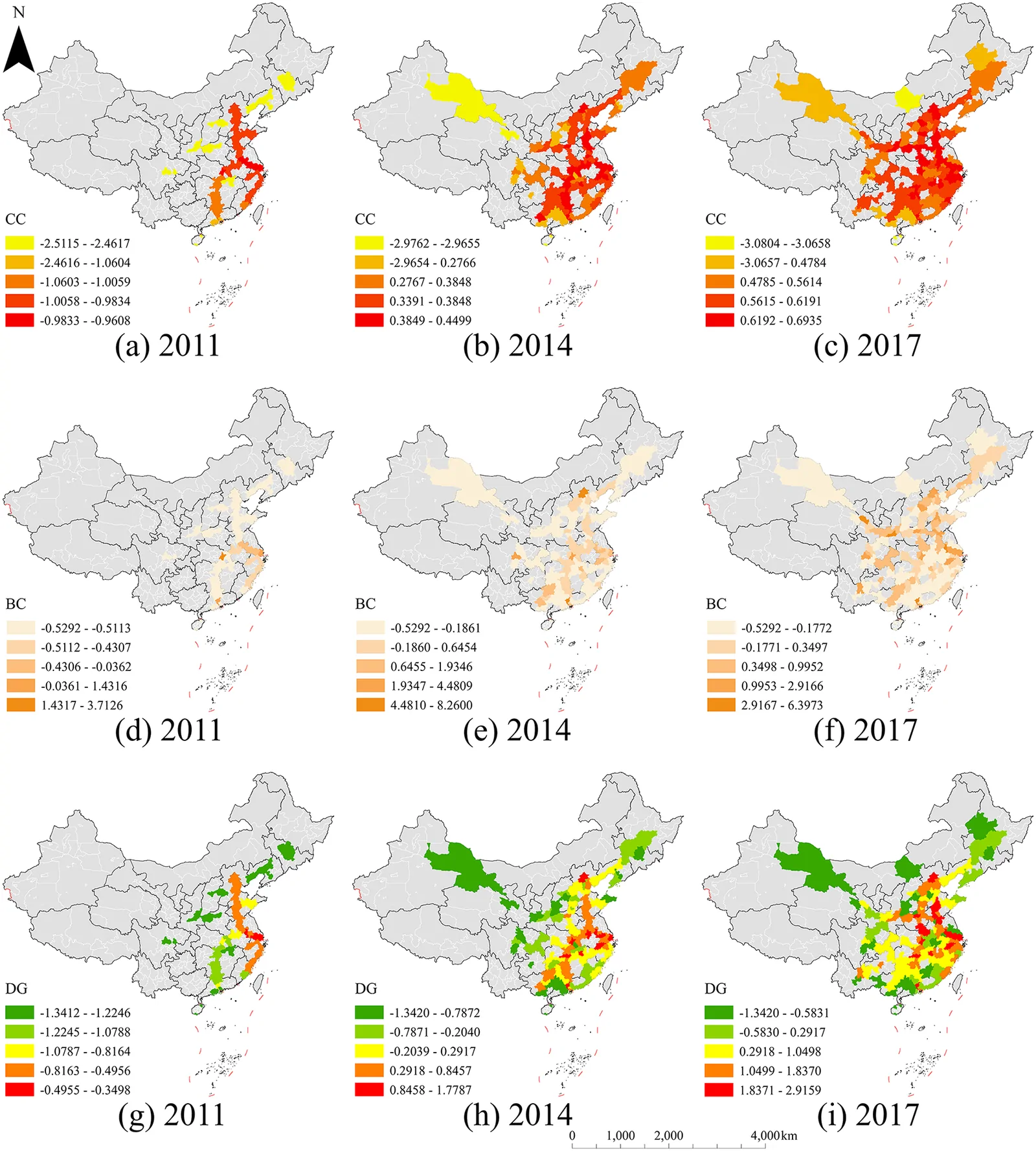
China HSR network centrality (CC, BC, and DG) in 2011, 2014, and 2017.

**Fig 5:**
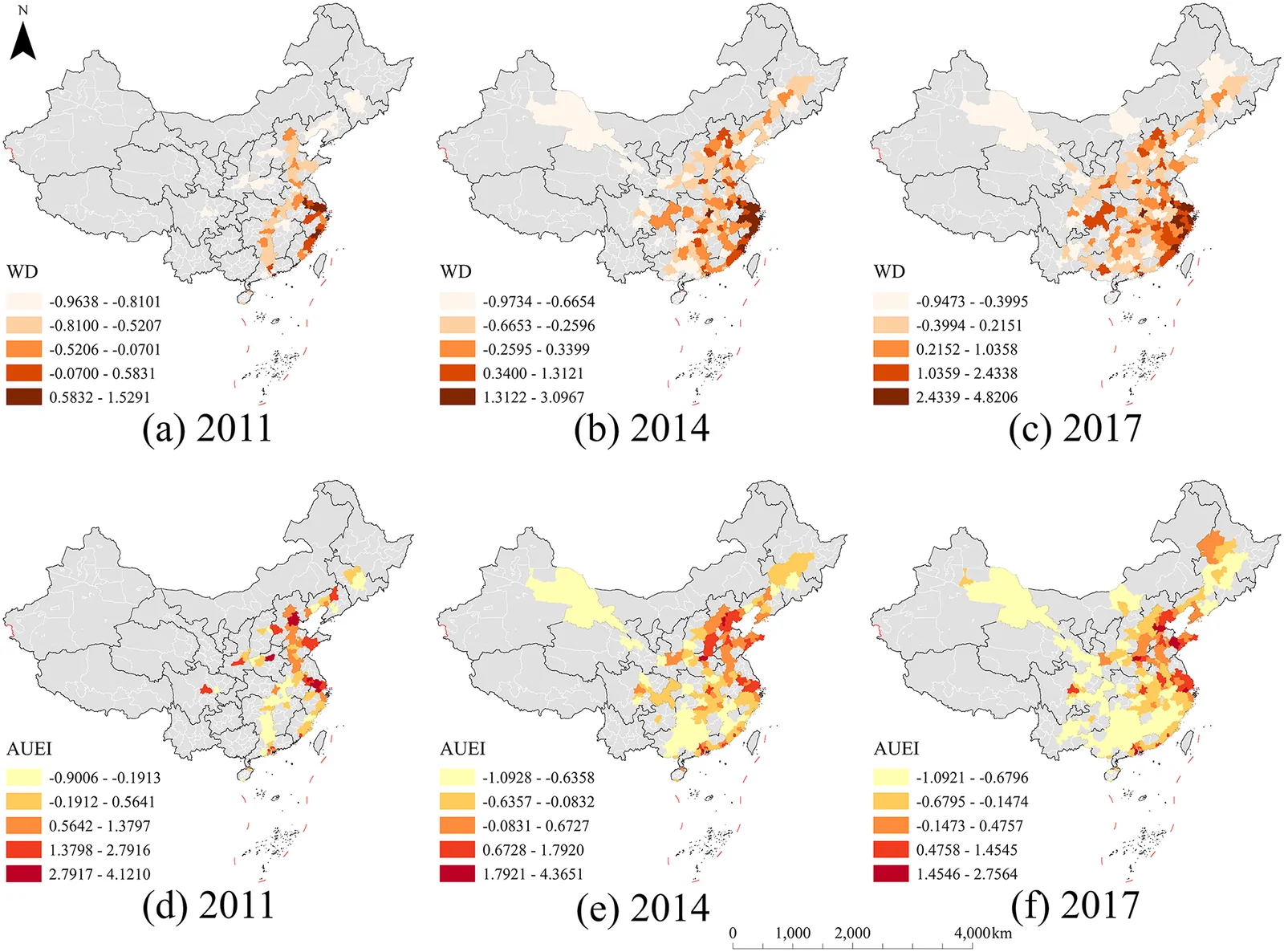
China HSR network centrality (WD) and AUEI in 2011, 2014, and 2017.

As can be seen from Figs 4 and 5, the highest nodes in the ranking of closeness centrality (in 2017) were Nanjing, Shanghai, Zhengzhou, Beijing, Wuhan and Wuxi. These cities have consistently ranked among the top, which reflects their positions at the main intersections of the high-speed rail corridor. From the perspective of intermediality and centrality, Guangzhou, Lanzhou and Xi ‘an are key resource transfer hubs and act as intermediaries for resource flow among regions. The top nodes arranged by degree centrality are Nanjing, Shanghai, Zhengzhou and Beijing, which have the most direct high-speed rail connections. From the perspective of urban structure trends, by 2017, the spatial distribution had evolved from the initial “axial corridor” model (concentrated along the Beijing-Guangzhou and Beijing-Shanghai lines) to a more “networked” structure, presenting a feature of “concentration and dispersion”: the status of core hub cities has been enhanced, and the connectivity of peripheral cities has significantly improved.

## Results

Subsequently, taking four variables related to HSR accessibility as explanatory variables, the indirect effects of these variables on urban expansion were examined through different mediating variables. According to the workflow given above, only the results are shown in Table 4, Table 5, Table 6, Table 7.

**Table 4:** Mediating effect of closeness centrality (CC) on urban expansion.

Variable	Total effect: CC→AUEI		Mediating variable: EMP	
			CC→EMP	CC+EMP→AUEI
CC	-0.1497*** (0.0396)		-0.0445* (0.0263)	-0.1383*** (0.0392)
Mediating variable				0.2575*** (0.0732)
Constant	0 (0.0185)		0 (0.0122)	0 (0.0182)
Control variable	Yes		Yes	Yes
City FE	Yes		Yes	Yes
Year FE	Yes		Yes	Yes
Obs.	418		418	418
Adj.R^2^	0.0652		0.1964	0.0902
F statistic	6.81		21.38	7.89
**Variable**	**Mediating variable: IRE**		**Mediating variable: TER**	
	**CC→IRE**	**CC+IRE→AUEI**	**CC→TER**	**CC+TER→AUEI**
CC	-0.0352* (0.0206)	-0.1441*** (0.0397)	0.0155 (0.0192)	-0.1567*** (0.0388)
Mediating variable		0.1600* (0.0945)		0.4541*** (0.0995)
Constant	0 (0.0096)	0 (0.0184)	0 (0.006)	0 (0.018)
Control variable	Yes	Yes	Yes	Yes
City FE	Yes	Yes	Yes	Yes
Year FE	Yes	Yes	Yes	Yes
Obs.	418	418	418	418
Adj.R^2^	0.579	0.0694	0.4956	0.1081
F statistic	115.69	6.18	82.93	9.42

Note: ***, **, and * represent significance at the 1%, 5%, and 10% levels, respectively. The total sample size is 418, the panel data covers three periods: 2011–2014, 2014–2017, and 2017–2020. The following tables (Tables 5–7-7) are all the same. The discrepancy between the sample size (418) and the number of cities (278) is due to the multi-observation unit processing for cities with multiple high-speed rail stations. The 278 cities serve as the core observational units of the study. Since some large and medium-sized cities (e.g., Beijing and Shanghai) have multiple high-speed rail (HSR) stations within their administrative boundaries (e.g., Beijing has Beijing Station and Beijing West Station, et al.), each HSR station constitutes an independent sample. Ultimately, this results in 418 valid samples.

**Table 5:** Mediating effect of betweenness centrality (BC) on urban expansion.

Variable	Total effect: BC→AUEI		Mediating variable: EMP	
			BC→EMP	BC+EMP→AUEI
BC	-0.0921** (0.0368)		0.0147 (0.0243)	-0.0963*** (0.0362)
Mediating variable				0.2848*** (0.0735)
Constant	0 (0.0186)		0 (0.0123)	0 (0.0183)
Control variable	Yes		Yes	Yes
City FE	Yes		Yes	Yes
Year FE	Yes		Yes	Yes
Obs.	418		418	418
Adj.R^2^	0.0473		0.1915	0.0786
F statistic	5.14		20.75	6.93
**Variable**	**Mediating variable: IRE**		**Mediating variable: TER**	
	**BC→IRE**	**BC+IRE→AUEI**	**BC→TER**	**BC+TER→AUEI**
BC	0.0806*** (0.0186)	-0.1122*** (0.0374)	-0.0015 (0.0176)	-0.0914** (0.036)
Mediating variable		0.2494** (0.0968)		0.437*** (0.1006)
Constant	0 (0.0094)	0 (0.0185)	0 (0.0089)	0 (0.0182)
Control variable	Yes	Yes	Yes	Yes
City FE	Yes	Yes	Yes	Yes
Year FE	Yes	Yes	Yes	Yes
Obs.	418	418	418	418
Adj.R^2^	0.5944	0.0601	0.4948	0.0868
F statistic	123.24	5.45	82.67	7.61

Note: ***, **, and * represent significance at the 1%, 5%, and 10% levels, respectively.

**Table 6:** Mediating effect of degree (DG) on urban expansion.

Variable	Total effect: DG→AUEI		Mediating variable: EMP	
			DG→EMP	DG+EMP→AUEI
DG	-0.2809*** (0.0542)		-0.0606* (0.0365)	-0.2658*** (0.0537)
Mediating variable				0.2498*** (0.0722)
Constant	0 (0.0182)		0 (0.0122)	0 (0.0179)
Control variable	Yes		Yes	Yes
City FE	Yes		Yes	Yes
Year FE	Yes		Yes	Yes
Obs.	418		418	418
Adj.R^2^	0.092		0.1962	0.1155
F statistic	9.45		21.35	10.08
**Variable**	**Mediating variable: IRE**		**Mediating variable: TER**	
	**DG→IRE**	**DG+IRE→AUEI**	**DG→TER**	**DG+TER→AUEI**
DG	0.0207 (0.0287)	-0.2852*** (0.054)	0.0475* (0.0265)	-0.3041*** (0.0529)
Mediating variable		0.2062** (0.0926)		0.4876*** (0.0979)
Constant	0 (0.0096)	0 (0.0181)	0 (0.0089)	0 (0.0177)
Control variable	Yes	Yes	Yes	Yes
City FE	Yes	Yes	Yes	Yes
Year FE	Yes	Yes	Yes	Yes
Obs.	418	418	418	418
Adj.R^2^	0.5765	0.1006	0.4987	0.1415
F statistic	114.54	8.78	83.95	12.46

Note: ***, **, and * represent significance at the 1%, 5%, and 10% levels, respectively.

**Table 7:** Mediating effect of FRE on urban expansion.

Variable	Total effect: FRE→AUEI		Mediating variable:EMP	
			FRE→EMP	FRE+EMP→AUEI
FRE	-0.1011 (0.0741)		-0.0328 (0.0486)	-0.0921 (0.073)
Mediating variable				0.2758*** (0.074)
Constant	0 (0.0187)		0 (0.0123)	0 (0.0184)
Control variable	Yes		Yes	Yes
City FE	Yes		Yes	Yes
Year FE	Yes		Yes	Yes
Obs.	418		418	418
Adj.R^2^	0.0371		0.1917	0.0664
F statistic	4.22		20.78	5.94
**Variable**	**Mediating variable:IRE**		**Mediating variable:TER**	
	**FRE→IRE**	**FRE+IRE→AUEI**	**FRE→TER**	**FRE+TER→AUEI**
FRE	0.2718*** (0.0357)	-0.1739** (0.0786)	-0.0102 (0.0353)	-0.0966 (0.0726)
Mediating variable		0.2678*** (0.1016)		0.4362*** (0.1012)
Constant	0 (0.009)	0 (0.0186)	0 (0.0089)	0 (0.0183)
Control variable	Yes	Yes	Yes	Yes
City FE	Yes	Yes	Yes	Yes
Year FE	Yes	Yes	Yes	Yes
Obs.	418	418	418	418
Adj.R^2^	0.6284	0.0508	0.4949	0.0765
F statistic	142.01	4.72	82.7	6.76

Note: ***, **, and * represent significance at the 1%, 5%, and 10% levels, respectively.

### Mediating effect of CC on urban expansion

Using CC as the explanatory variable, and EMP, IRE, and TER as mediating variables, successive mediating mechanism tests were conducted with the results shown in Table 4 (numbers in parentheses represent standard deviations; same for Tables 5‒7 below). The second column in Table 4 records the total effect of CC on urban expansion, with a coefficient of −0.1497 (p < 0.01), indicating that an increase in HSR network CC can significantly inhibit extensive urban outward expansion.

When the mediating variable was EMP, the regression coefficient of CC on EMP is −0.0445, and the regression coefficient of EMP on urban expansion is 0.2575. After adding EMP, the direct effect coefficient of CC on urban expansion is −0.1383, all of which pass the significance test. This indicates that an increase in CC will reduce EMP, and a decrease in EMP will in turn inhibit urban expansion. Therefore, CC can further inhibit urban expansion by reducing the degree of employment agglomeration. Since the direction of the indirect effect is consistent with the direct effect, EMP plays a partial mediating role in this process.

When the mediating variable was IRE, the regression coefficients of CC on IRE is −0.0352, and the regression coefficient of IRE on urban expansion is 0.1600. After adding IRE, the direct effect coefficient of CC on urban expansion becomes −0.1441, all of which pass the significance test. This indicates that an increase in CC inhibits IRE, thereby indirectly reducing urban expansion. Since the direction of the indirect effect is consistent with the direct effect, IRE plays a partial mediating role in this process.

When the mediating variable was TER, the regression coefficients of CC on TER is 0.0155, which fails to pass the significance test, indicating that the mediating effect of TER is not significant. This implies that the enhancement of HSR closeness centrality does not significantly act on overall urban expansion by influencing industrial structure upgrading.

### Mediating effect of BC on urban expansion

The mediating mechanism of BC on urban expansion was tested using EMP, IRE and TER, with the results are shown in Table 5. The second column in Table 5 reports the total effect of BC on urban expansion, with a coefficient of −0.0921, passing the 5% significance test, indicating that the enhancement of urban hub control capability helps guide compact spatial development.

When the mediating variable is EMP, the regression coefficient of BC on EMP is 0.0147, which does not pass the significance test; therefore, the mediating effect of EMP is not significant.

When IRE served as the mediating variable, the regression coefficients of BC on IRE is 0.0806, and the regression coefficient of IRE on urban expansion is 0.2494. After adding IRE, the direct effect coefficient of BC on urban expansion becomes −0.1122, all of which pass the significance tests. This indicates that an increase in BC can significantly promote IRE, and IRE will further promote urban expansion. Therefore, although the enhancement of HSR hub status overall inhibits urban expansion, it also forms a certain expansion effect by stimulating real estate investment at the same time. Since the indirect effect is positive while the direct effect is negative, and their directions are opposite, investment in real estate development exhibits a masking effect. The size of the masking effect is 17.92%, meaning it weakens the negative impact of BC on urban expansion.

When TER was the mediating variable, the regression coefficient of BC on TER is −0.0015, which fails to pass the significance test; thus, the mediating effect of TER is not significant.

### Mediating effect of DG on urban expansion

Using DG as the explanatory variable, and EMP, IRE and TER, successive mediating mechanism tests were conducted with the results were shown in Table 6. The second column is the total effect of DG on urban expansion, with a coefficient of −0.2809, passing the 1% significance test, indicating that the higher the degree of HSR network connection, the more significantly it can inhibit urban expansion, which shows that the enhancement of HSR connections helps optimize spatial resource allocation and improve urban land development efficiency.

When the mediating variable is EMP, the regression coefficient of DG on EMP is −0.0606, and the regression coefficient of EMP on urban expansion is 0.2498. After adding EMP, the direct effect coefficient of DG on urban expansion is −0.2658, all of which pass the significance test. This indicates that an increase in DG will reduce local EMP, which further inhibits disorderly urban expansion due to the slowdown of employment agglomeration. Since the indirect effect (negative) is consistent in direction with the direct effect (negative), employment density plays a partial mediating role in this process.

When the mediating variable was IRE, the regression coefficients of DG on IRE is 0.0207, which does not pass the significance test; therefore, the mediating effect of IRE along this path is not significant. This indicates that the elevation of degree centrality does not significantly affect urban expansion through the real estate investment path.

When the mediating variable was TER, the regression coefficients of the DG on TER is 0.0475, and the coefficient of TER on urban expansion is 0.4876. After adding TER, the direct effect coefficient of DG on urban expansion is −0.3041, all of which pass the significance test. This shows that an increase in DG significantly promotes the proportion of added value of the tertiary industry, and the growth of TER will further stimulate the land demand for urban expansion. Since the indirect effect is positive while the direct effect is negative, and their directions are opposite, the proportion of the tertiary industry exhibits a masking effect. The size of the masking effect is 7.62%, meaning that the land demand from industrial structure upgrading to some extent weakens the overall inhibitory effect of degree centrality on urban sprawl.

### Mediating effect of FRE on urban expansion

Using FRE as the explanatory variable and EMP, IRE and TER as mediating variables, the results are shown in Table 7. The second column shows the total effect of FRE on urban expansion, with a coefficient of −0.1011, which does not pass the significance test. Although the total effect is not significant, the direct and indirect effects might cancel each other out due to opposite signs, necessitating further examination of each path mechanism.

When the mediating variable is EMP, the regression coefficient of FRE on EMP (−0.0328) fails to pass the significance test, and the mediating effect of EMP is not significant.

When the mediating variable was IRE, the regression coefficients of FRE on IRE is 0.2718, and the regression coefficient of IRE on urban expansion is 0.2678. After adding IRE, the direct effect coefficient of FRE on urban expansion becomes −0.1739, all of which pass the significance tests. This indicates that an increase in HSR stop frequency can significantly boost local IRE, and the boom in IRE will further promote overall urban expansion. Since the indirect effect is strongly positive and the direct effect is negative, they move in opposite directions and cause the total effect to be canceled out to insignificance, representing a typical masking effect. The size of the masking effect is as high as 41.86%, meaning that the spatial expansion dividend of real estate prosperity brought by HSR largely covers up and cancels out the inhibitory effect of HSR network centripetal agglomeration on urban peripheral sprawl.

When the mediating variable is TER, the coefficient of FRE on TER (−0.0102) fails to pass the significance test, and the mediating effect of TER is not significant.

### Robustness Test: Heterogeneity boundaries and interaction effects

To further reveal the deep impact boundaries of HSR network characteristics on urban spatial expansion and test the heterogeneous performance of the previously identified mediating paths under different conditions, this study further introduces interaction terms between the explanatory variables and core mediating variables into the two-way fixed effects model for verification. The regression results are presented in Table 8.

**Table 8:** Robustness check: Interaction effects between independent and mediating variables.

Variable	(1) AUEI	(2) AUEI
Interaction term	CC × EMP	FRE × IRE
X	−0.1531*** (0.0398)	−0.1658** (0.0776)
Mediating variable	0.5054*** (0.1437)	0.2924*** (0.1006)
Interaction	−0.7297** (0.3644)	−0.2082*** (0.0612)
Control variable	Yes	Yes
City FE	Yes	Yes
Year FE	Yes	Yes
Obs.	418	418
Adj.R^2^	0.0969	0.0747
F statistic	7.39	5.81

Note: ***, **, and * represent significance at the 1%, 5%, and 10% levels, respectively.

Model (1) shows the interaction effect results of CC and EMP. The coefficient of CC on urban expansion is −0.1531, the impact of EMP on urban expansion is 0.5054, and the coefficient of the interaction term is −0.7297, all of which pass the significance test. This result indicates that EMP plays a significant negative regulatory role in the inhibitory effect of CC on urban expansion. Specifically, when a city has a high CC in the HSR network, better accessibility will accelerate the agglomeration of production factors around the hub. At this time, if the city’s EMP is already relatively high, the employment agglomeration brought by the HSR network will be more intense, prompting enterprises and labor forces to lean toward high-density, vertical spatial utilization within the city, thereby further reinforcing the inhibitory effect of HSR on extensive urban outward sprawl. In other words, in cities with highly concentrated employment, the elevation of the HSR network hub position can more effectively drive the transformation of the city toward a “compact” spatial structure.

Model (2) displays the interaction effect results of FRE and IRE. The coefficient of FRE on urban expansion is −0.1658, the impact of IRE on urban expansion is 0.2924, and the interaction term coefficient is −0.2082, all of which pass the significance test. This strongly demonstrates that with the densification and maturation of a city’s FRE, the powerful agglomeration effect generated by the HSR network can effectively weaken and mitigate the extensive sprawl tendency brought by IRE. In cities with frequent HSR frequencies and extremely strong hub effects, real estate capital prefers high-plot-ratio TOD (Transit-Oriented Development) comprehensive development around HSR stations rather than blindly expanding at the urban periphery. Therefore, the enhancement of HSR network density not only possesses a spatial convergence effect itself but can also reverse the negative drive of real estate investment on urban peripheral sprawl by optimizing the spatial configuration pattern of capital.

## Discussion

### Influence mechanism of HSR accessibility on urban expansion

First, network centrality exerts an overall inhibitory effect on urban expansion. The regression results demonstrate that the total effects of the three HSR network centrality indicators (CC, BC, and DG) on urban expansion are all significantly negative, which indicates that the higher a city’s topological position within the national HSR network, the more pronounced its spatial intensification becomes. High centrality accelerates cross-regional factor flows through space-time compression; when internal urban development matures, the blind flow of factors from the center to the periphery is curbed, constraining disorderly urban peripheral land expansion as a whole.

Second, the mediating variables present distinct transmission and regulatory boundaries. EMP features a mechanism of reinforcing agglomeration. Empowered by the HSR network, labor further clusters and undergoes vertical restructuring around stations, amplifying the inhibition of urban expansion. Conversely, IRE exhibits a strong masking effect: an increase in FRE stimulates the growth of IRE, concealing the compact trend. However, along with the maturation of the HSR network, it can significantly constrain the blind expansion of real estate capital into cities, guiding real estate to shift toward high-plot-ratio TOD complexes around transportation hubs. Furthermore, the elevation of HSR centrality does not significantly alter TER, implying that HSR’s shaping of urban form relies more on factors with spatial characteristics rather than simple transformations of the macro industrial structure.

### Policy implications

The result of “HSR centrality and frequency inhibit urban expansion” obtained in this study reflects the Chinese government’s control and adjustment of land use during the city’s transition from “incremental expansion” to “stock intensification.”

HSR network centrality possesses a powerful structural containment capability over urban expansion, and the government should fully unleash hub dividends. Especially in core node cities with high network positions, blind and unnecessary planning of peripheral new towns and development zones should be strictly restricted, commercial, business, and public services should be guided to gather around stations, and high-intensity TOD comprehensive development should be conducted around HSR stations, converting transportation nodes into anchors of comprehensive urban economic vitality.

While the increase in HSR shifts boosts real estate investment and drives spatial sprawl, local governments should carefully evaluate the short-term benefits brought by “land finance.” The increase in HSR frequency should be combined with total land supply restrictions to guide real estate capital away from low-density expansion toward multi-functional, vertically intensive development.

Combining the interaction effect results, relevant industrial and infrastructure resources should be preferentially tilted toward node cities with a certain labor foundation, so as to take the lead in achieving a benign transformation of HSR dividends and maximizing the policy spillover effect of intensive and economical spatial utilization.

### Future work

In this study, we explored the impact mechanism of multi-dimensional accessibility in the HSR network on urban expansion, but the selection of mediating variables presents certain limitations. Changes in HSR accessibility influence the flow of innovation factors, and shifts in innovation capacity can subsequently impact urban expansion. Future research should incorporate relevant indicators of scientific and technological innovation, such as the number of patents granted, investments in scientific research and development, and urban innovation vitality. Meanwhile, longer time series data can be introduced in the future to precisely calculate the non-linear characteristics of HSR’s inhibitory effect on urban expansion at different developmental stages and under different scale thresholds.

## Conclusion

Based on the panel data of 278 prefecture-level cities in China in 2011, 2014, and 2017, this paper constructs mediating effect and interaction effect models to systematically reveal the impact of HSR network characteristics on the intensity of urban spatial expansion and the mediating regulatory paths. The research indicates that:

1. HSR network centrality (CC, BC, DG) constitutes a structural “brake valve” that overall inhibits extensive and disorderly urban expansion. The “opening promotes sprawl” effect identified in traditional literature based on dummy variables has given way to the “centripetal agglomeration” and intensive convergence brought by high centrality during the network deepening period [38].
2. This inhibitory path is regulated by the dual boundary conditions of internal employment density and external capital flows. A high level of employment density can effectively undertake and amplify the centripetal pull of centrality; although the increase in HSR frequency induces masking expansion through the mediating channel of real estate investment in the short term, as the operational network advances toward maturity, high-frequency shifts can significantly constrain and reverse the sprawl tendency of real estate investment through efficient interaction with capital.

In conclusion, as China’s HSR moves from “single-line opening” to “deep networking,” its spatial effect is undergoing a fundamental shift, having become a key spatial driving force promoting the transition of Chinese urban land factors toward intensive, compact, and stock-based development.

## Supporting information

S1 Fig China’s HSR network in 2011, 2014, and 2017. (TIF)

S2 Fig China’s HSR flows in 2011, 2014, and 2017. (TIF)

S3 Fig Mediating-effect model conceptual figure. (TIF)

S4 Fig China HSR network centrality (CC, BC, and DG) in 2011, 2014, and 2017. (TIF)

S5 Fig China HSR network centrality (WD) and AUEI in 2011, 2014, and 2017. (TIF)
